# *KmerAperture*: Retaining *k*-mer synteny for alignment-free extraction of core and accessory differences between bacterial genomes

**DOI:** 10.1371/journal.pgen.1011184

**Published:** 2024-04-29

**Authors:** Matthew P. Moore, Mirjam Laager, Paolo Ribeca, Xavier Didelot

**Affiliations:** 1 School of Life Sciences, University of Warwick, Coventry, United Kingdom; 2 Department of Statistics, University of Warwick, Coventry, United Kingdom; 3 Division of Transplant Immunology and Nephrology, University Hospital Basel, Basel, Switzerland; 4 UK Health Security Agency, London, United Kingdom; Case Western Reserve University, UNITED STATES

## Abstract

By decomposing genome sequences into *k*-mers, it is possible to estimate genome differences without alignment. Techniques such as *k-*mer minimisers, for example MinHash, have been developed and are often accurate approximations of distances based on full *k*-mer sets. These and other alignment-free methods avoid the large temporal and computational expense of alignment. However, these *k*-mer set comparisons are not entirely accurate within-species and can be completely inaccurate within-lineage. This is due, in part, to their inability to distinguish core polymorphism from accessory differences. Here we present a new approach, *KmerAperture*, which uses information on the *k*-mer relative genomic positions to determine the type of polymorphism causing differences in *k*-mer presence and absence between pairs of genomes. Single SNPs are expected to result in *k* unique contiguous *k*-mers per genome. On the other hand, contiguous series > *k* may be caused by accessory differences of length *S*-*k*+1; when the start and end of the sequence are contiguous with homologous sequence. Alternatively, they may be caused by multiple SNPs within *k* bp from each other and *KmerAperture* can determine whether that is the case. To demonstrate use cases *KmerAperture* was benchmarked using datasets including a very low diversity simulated population with accessory content independent from the number of SNPs, a simulated population where SNPs are spatially dense, a moderately diverse real cluster of genomes (*Escherichia coli* ST1193) with a large accessory genome and a low diversity real genome cluster (*Salmonella* Typhimurium ST34). We show that *KmerAperture* can accurately distinguish both core and accessory sequence diversity without alignment, outperforming other *k*-mer based tools.

## Author summary

The *KmerAperture* algorithm provides a substantive progression in alignment-free methodologies in bacterial comparative genomics. The utility of bacterial genomes for epidemiology and uncovering the genetic basis for phenotypic diversity and adaptation lies in our ability to compare them at scale. Perhaps the most limiting feature of genetic analysis workflows is sequence alignment. A number of *k*-mer based alignment-free methodologies have been developed to avoid the temporal and compute cost of alignment. However, in closely related bacteria the signal of polymorphisms, as they are represented by unique *k*-mers, is lost to even a small amount of unshared sequence, whether real or artefactual. Here, we show that it’s possible to discern SNP diversity without alignment, including when SNPs are within *k* of one another, a perennial problem for *k*-mer analyses.

## Introduction

The increased availability of whole genome sequencing data provides the opportunity to transform bacterial population genetics and infectious disease epidemiology [1,2]. The United Kingdom Health Security agency, for example, now conducts whole genome sequencing and SNP-distance based typing as routine [3]. Even once the labour of collection and sequencing of thousands of bacterial is complete, the challenges of bioinformatic analysis are not trivial. The dynamic comparison of sequences with alignment algorithms remains temporally and computationally expensive. It is with these relative differences that detailed comparative and phylogenetic analysis is possible. Comparison of exclusive pairs of 5,000 genomes for instance scales quadratically with >12 million alignments. Fortunately, we can shortcut this to 5,000 alignments by aligning each to an appropriate reference [1,4]. With hundreds of thousands of genomes now available for some species [5], even linear scaling risks becoming infeasible [1].

A number of efficient alignment-free approaches have been developed to circumvent this issue. The most efficient of these involve decomposition of genomes into *k*-mers (*k*-mers/kmers/*n*-grams; unique sequences of length *k*), subsampling hashed *k*-mers into ‘sketches’ and performing set analysis [6–9]. Methods such as the min-wise independent permutations local sensitivity hashing scheme (MinHash), accurately approximate the full *k*-mer set differences across genomes and are extremely fast [6]. Whilst these approaches can successfully determine distances between genomes accurate enough to differentiate between natural populations within a bacterial species, comparable to multi locus sequence types (MLST), within-population estimates are inaccurate. Ultimately, this is due to the inability to know whether mismatching *k*-mers result from polymorphism or relative presence/absence of the sequence.

Set-based *k*-mer comparisons, including full sets, can become increasingly inaccurate for bacterial genomes the lower the SNP diversity and it is these highly similar genomes that are those that are most relevant for outbreak detection and further analysis for public health [10–12]. The inaccuracy is due to the presence of unshared sequence in sequenced genomes even in the those with very little real diversity (very few SNPs). This confounds SNP distance estimation by *k*-mer sets as the unshared sequence represented by mismatching *k*-mers may increase in relative terms. Distance estimates from *k*-mers will be driven by even a small amount of unshared sequence, whether real or artefactual [13]. SNPs at greater than *k* bp apart will generate the same number of unique *k*-mers (*U*) per genome in a pair as SNPs between them. An unshared sequence need only be of *L* > 2(*k*+*U*-1), where *L* is non-contiguous sequence length. For instance, *k* = 11 and 5 SNPs in a genome pair will generate 110 unique *k*-mers, such that *L*>130 would be required to generate more unique *k*-mers. For low SNP diversity and even for re-sequenced (identical bacterial genome assemblies) there can be large stretches of unshared sequence and relatedness will be substantially underestimated [13].

Inspired by molecular probes, the concept of the split *k*-mer was introduced by saSNP [14], which became kSNP [15,16]. The kSNP algorithm first counts *k*-mers with Jellyfish [17] and constructs a suffix tree. Candidate SNPs are then gathered from local *k*-mers in the suffix tree before being aligned back to the genomes to regain their genomic context. The middle based of odd length *k*-mers is then allowed to vary. Split *k*-mer Analysis (SKA) builds upon the split *k*-mer concept with a focus on small genomes. With small genomes, such as bacterial genomes, there is no need for the memory efficiency of the suffix tree and associated temporal overhead. The split *k*-mer concept is predicated on the fact that as odd-numbered *k*-mers are generated from respective homologous regions, SNPs can be accounted for where the middle base varies. Each SNP will be represented by a split *k*-mer. Performance in accuracy benchmarking is similar between kSNP and SKA [18,19]. Both kSNP and SKA however, will not gather SNPs that are close together on the genome. Specifically, those which are within a half split *k*-mer ((< (*k*-1) / 2) apart)[15]. Both approaches, however, discern for many mismatching *k*-mers whether they result from unshared sequence or base variation. Future approaches to scalable comparison may involve exact *k*-mer or split *k*-mer matching against a previously determined SNP panel [20].

Here, we present *KmerAperture*, a novel alignment-free algorithm with the ability to determine bacterial genetic differences without alignment. Mismatching *k*-mers are linked to their original synteny, taking advantage of initial efficient *k*-mer set analysis to generate the relative *k*-mer complements and exclude the intersection. Contiguous *k*-mer series may then be investigated as to whether they are generated by presence/absence of sequences, SNPs or repeats. Crucially, *KmerAperture* is also able to gather SNPs within *k* of one another or (*k*-1) / 2 on the genome, a confounding feature of *k*-mer and split *k*-mer comparisons.

## Results

### The *KmerAperture* algorithm

**Fig 1:**
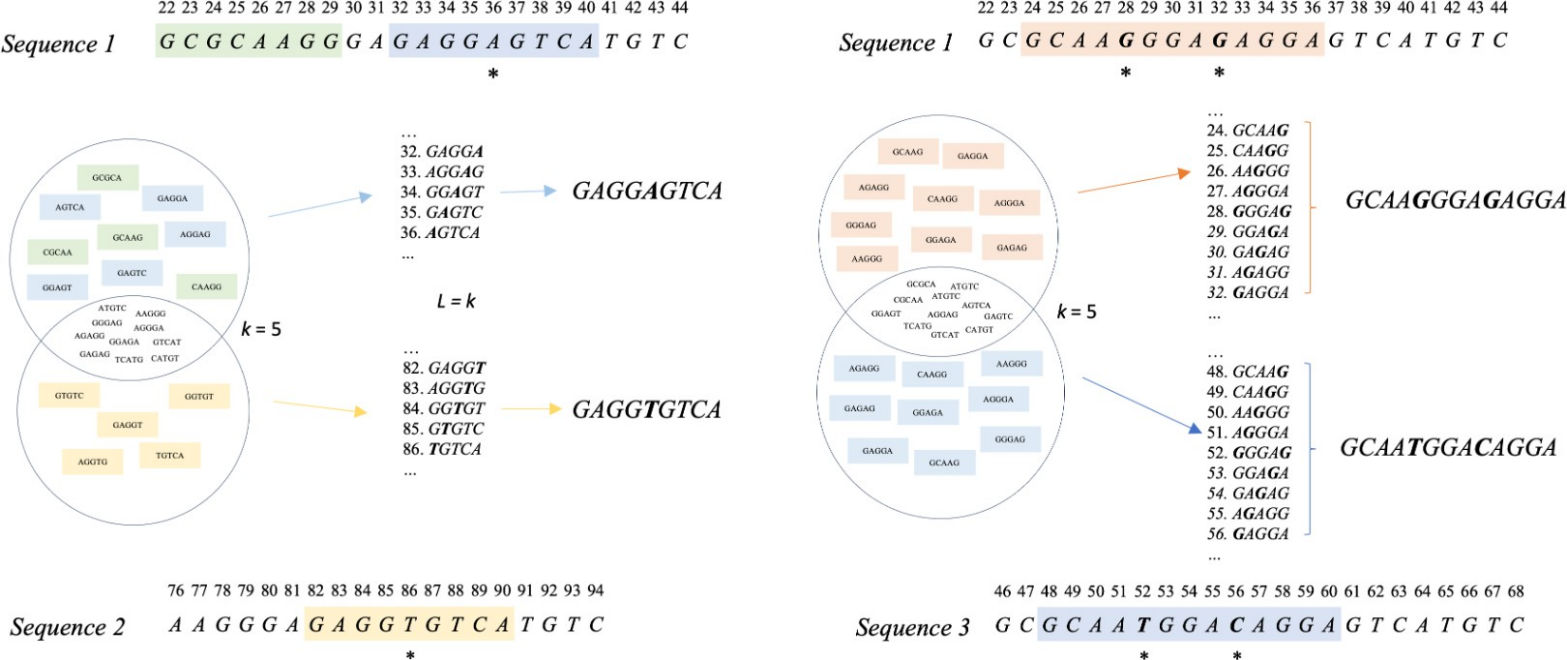
A toy example of two pairwise genome comparisons with *KmerAperture* is demonstrated, with *k* = 5. Three possibilities are illustrated: A SNP, unshared sequence or SNPs within *k* of one another. For each sequence, shaded regions show the total sequence that will be contained within unique *k*-mers. Sets are unordered so initially their synteny is lost, but regained by mapping back to the original, enumerated *k*-mer list. SNPs are denoted by a * beneath the base. Between Sequence 1 (S1) and Sequence 2 (S2), ten *k*-mers are shared, whilst there are 9 unique to S1 and 5 unique to S2. The sequences are different lengths and with different relative start positions. Between S1 and Sequence 3 (S3), the relative start positions also differ and there are SNPs spaced by 3bp. As a result, 5-mers would include both SNPs, rather than generating *k* unique *k*-mers per SNP. Nonetheless, where the original sequence is reconstructed and where reconstructed sequence lengths are equivalent, mismatches counted for all pairs.

The algorithm compares pairs of genomes by subtracting sets of *k*-mers before mapping unique *k*-mers back to their respective enumerated lists. In doing so we are able to examine those contiguous series of *k*-mers (*L*) likely to be generated by polymorphism. We’ve implemented this as reference-based so that the scaling is linear, rather than quadratic and so that a pairwise pseudoalignment is output for downstream analysis with standardised absolute positions. Estimated accessory boundaries are also output. We propose that our contiguous *k*-mer model, which takes advantage of the efficiency of *k*-mer set analysis by eliminating the majority of *k*-mers which are exact matches, may be adopted in alignment-free frameworks. Fig 1A provides a toy example of the *KmerAperture* algorithm. Sequence 1 and Sequence 2 are separated by a single SNP and a further 4bp sequence present only in Sequence 1. The sequences are decomposed into *k*-mer sets where *k* = 5 and their relative complements determined. We can anticipate that a SNP in otherwise shared sequence will generate *k* unique *k*-mers per set. As such, we can expect series where *L* = *k* to have been generated by a SNP. For all such series, the middle *k*-mer is extracted and an all-against-all comparison conducted for those differing by only the middle base.

When there is an isolated unshared sequence of length *L* we can anticipate this to produce *L-k+*1 unique *k*-mers covering the unshared sequence. In the case, as in Fig 1A, that the unshared sequence is also contiguous with shared sequence it will generate a further *k-*1 unique *k*-mers for each contiguous side. Alternatively, we anticipate a SNP to generate *k* unique *k*-mers in each set except when the SNP is within *k* of another SNP or sequence start or end (such as the end of a contig).

In Fig 1, *KmerAperture* would correctly discern mismatched *k*-mers resulting from SNPs vs accessory sequence. Both set complements would result in contiguous *k*-mers of serial length *k*. As a result, the mean estimated SNPs would be calculated and it would be determined that in the comparable sequence regions there is a single SNP. *KmerAperture* would additionally discern, from the five contiguous *k*-mers unique to the query sequence, that there was an accessory difference of 4bp (since these *k*-mers are contiguous with shared sequence on one side, they are expected to create (*L*-*k*+1)+(*k*-1) = *L k*-mers). For SNPs within *k* of another on the genome, they will produce series of length *L*>*k*+2 up to a maximum of *S*(*k*-1)+1 where *S* is the number of SNPs within *k* of one another.

### Rationale for datasets

To demonstrate its usefulness in a number of relevant use cases, we benchmarked *KmerAperture* in four scenarios: a very low diversity simulated population with accessory content independent from the number of SNPs; a simulated population where SNPs are spatially dense; a moderately diverse real cluster of genomes (*Escherichia coli* ST1193) with a large accessory genome; and a low SNP diversity real genome cluster (*Salmonella* Typhimurium ST34).

### Application to simulated genomes

The first simulated genome set includes 500 genomes with varying degrees of accessory difference to the reference and a range of 1–100 SNPs (median 11 SNPs). *KmerAperture* estimated a median (range) of 11 (0–100) SNPs with *k* = 19, 25 and 31. SKA produced the same median (range) of 11 (0–100) across all sizes of *k*.

The ability of *KmerAperture* and SKA to identify SNP counts less than or equal to various thresholds was assessed. *KmerAperture* produced high positive predictive values and specificity across SNP thresholds of ≤25,20,15,10 and 5. Across all choices of *k* and all thresholds with *KmerAperture* there were no false negatives (no overprediction of SNP values above a threshold), meaning sensitivity was 100% in all cases. There was a small number of false positives with between 2 and 5 for *k* = 19 and 1–3 for *k* = 31. With SKA there was a similarly small number of false positives with a maximum of 4 SNPs at *k* = 19 and a threshold of ≤15 SNPs. With SKA the specificity and PPV was equivalent or greater in all cases. However, there was a single false negative with *k* = 19 and 25 for the ≤5 SNP threshold (S2 Table).

For *k* = 19, 25 and 31, *KmerAperture* estimated the accessory difference for each genome compared with the reference. Relative presence was estimated for each genome in each pair. In all cases, it was correctly estimated that there was no relative additional sequence in the reference genome (0bp estimated). The relative additional query sequence ranged from a total size of 4,139bp to 1,926,407bp with a median of 736,708. This included 169 genomes with >1Mbp additional sequence. *KmerAperture* also attempts to identify these regions and extract them. The median absolute error as a percent of bp different to the real accessory size was 0.0017%, with a maximum error of 27.9% (of 827,256bp accessory).

In the following, we define *k*-*clustered SNPs* as consecutive SNPs along the genome spaced by no more than *k*-1. As a window of length *k* slides along by a single base, SNPs with ≤*k-*2 bases between them may be included in the same window. Greater than two SNPs may then also ‘chain’ together in a *k*-cluster.

Three genome sets were simulated with 50, 100 or 150 SNPs (n = 750 genomes total). Each set contained 250 genomes evenly divided (n = 50 genomes) into those with 20%, 40%, 60%, 80% and 100% of their SNPs being clustered within *k* = 25 of one another. It was randomly determined how many of the SNPs would form these SNP chains, for instance 50 SNPs being 100% *k*-clustered could involve three *k*-clusters of size 10, 10 and 30 SNPs within *k* of on another. Detecting *k*-clusters is a major weakness of *k*-mer based methods and we expect a degree of non-retrieval in these test conditions. Performance was better for *KmerAperture* than for SKA in the retrieval of SNPs and *k*-clustered SNPs, across all densities (20%-100%) and overall SNP number (50, 100 and 150 SNPs).

**Fig 2:**
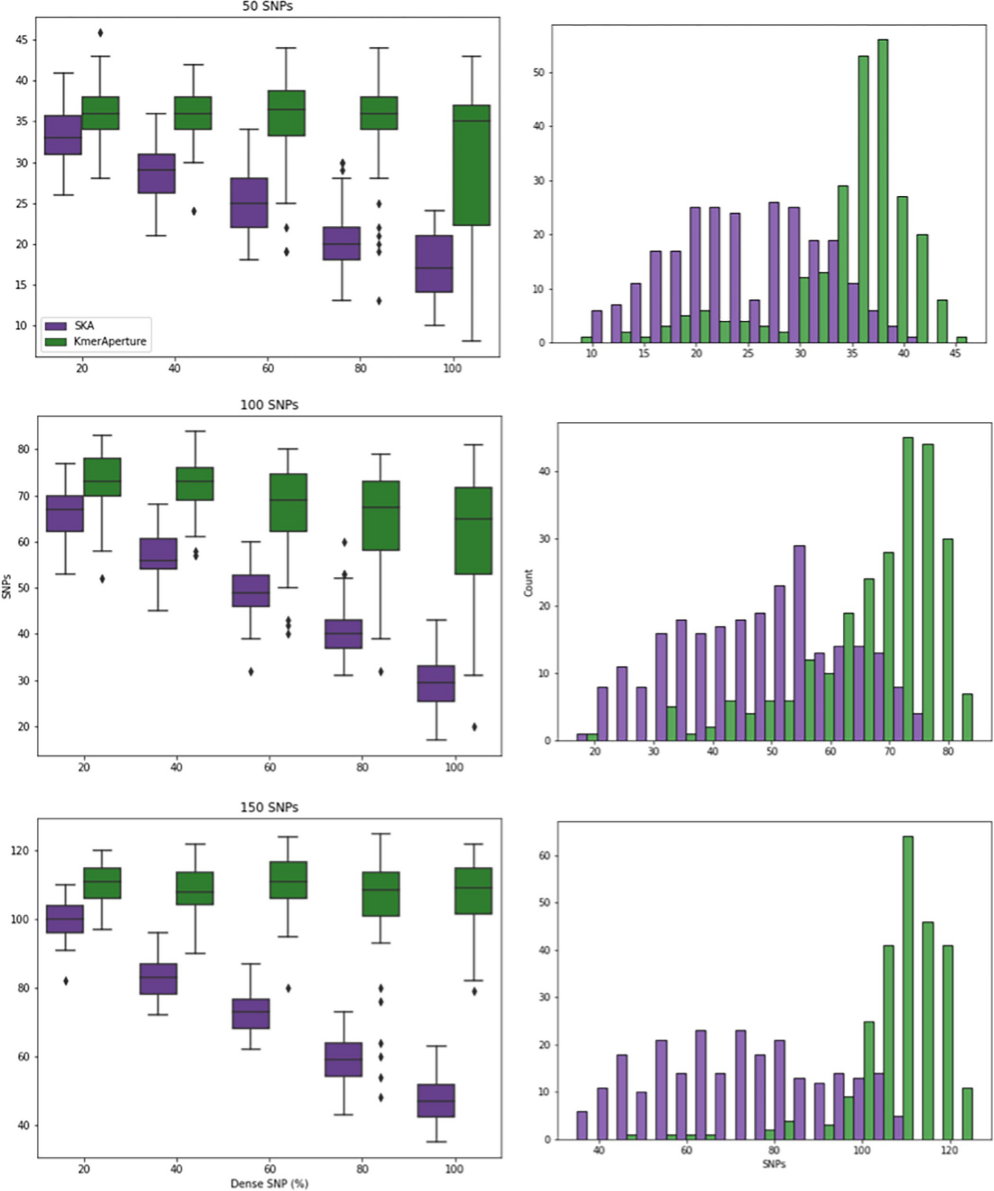
Boxplots (left) showing the range and median SNPs recovered by SKA (purple) and *KmerAperture* (green). Upper to lower are datasets with 50, 100 and 150 SNPs respectively. Each boxplot represents 250 genome SNP values, split evenly across 20%, 40%, 60%, 80% and 100% of SNPs that are *k*-clustered. Number of chains are randomly determined. The *k*-clustering is with *k* = 25. On the right, histograms showing the distribution of SNPs recovered by SKA or *KmerAperture* for all 250 genomes from 20–100% *k*-clustered SNPs.

The median reduction in retrieval performance with increasing *k*-clustering was smaller for *KmerAperture*. There was a relative decrease of SNPs retrieved from 20% to 100% fraction of 2.78%, 10.95% and 1.8% for 50, 100 and 150 SNPs respectively. For comparison, the relative reduction in performance with SKA between 20% and 100% of *k*-clustered SNPs was 48.48%, 55.97% and 53% for 50, 100 and 150 SNPs respectively. The range in performance increased for *KmerAperture* with increased *k*-clustering with minimum retrieval as low as 8/50 SNPs and 20/100 SNPs retrieved at 100% *k*-clustering and 48/150 SNPs retrieved at 80% *k*-clustering. There was a greater range in performance with *KmerAperture*, whilst SKA had a greater minimum retrieval in two categories (50 SNPs, 100% *k*-clustering and 100 SNPs, 20% *k*-clustering). In the remaining 13/15 the greater minimum retrieval was with *KmerAperture*. In all categories the greatest median and max retrieval was with *KmerAperture* (Fig 2 and S2 Table).

### Application to a cluster of *Escherichia coli* genomes

**Fig 3:**
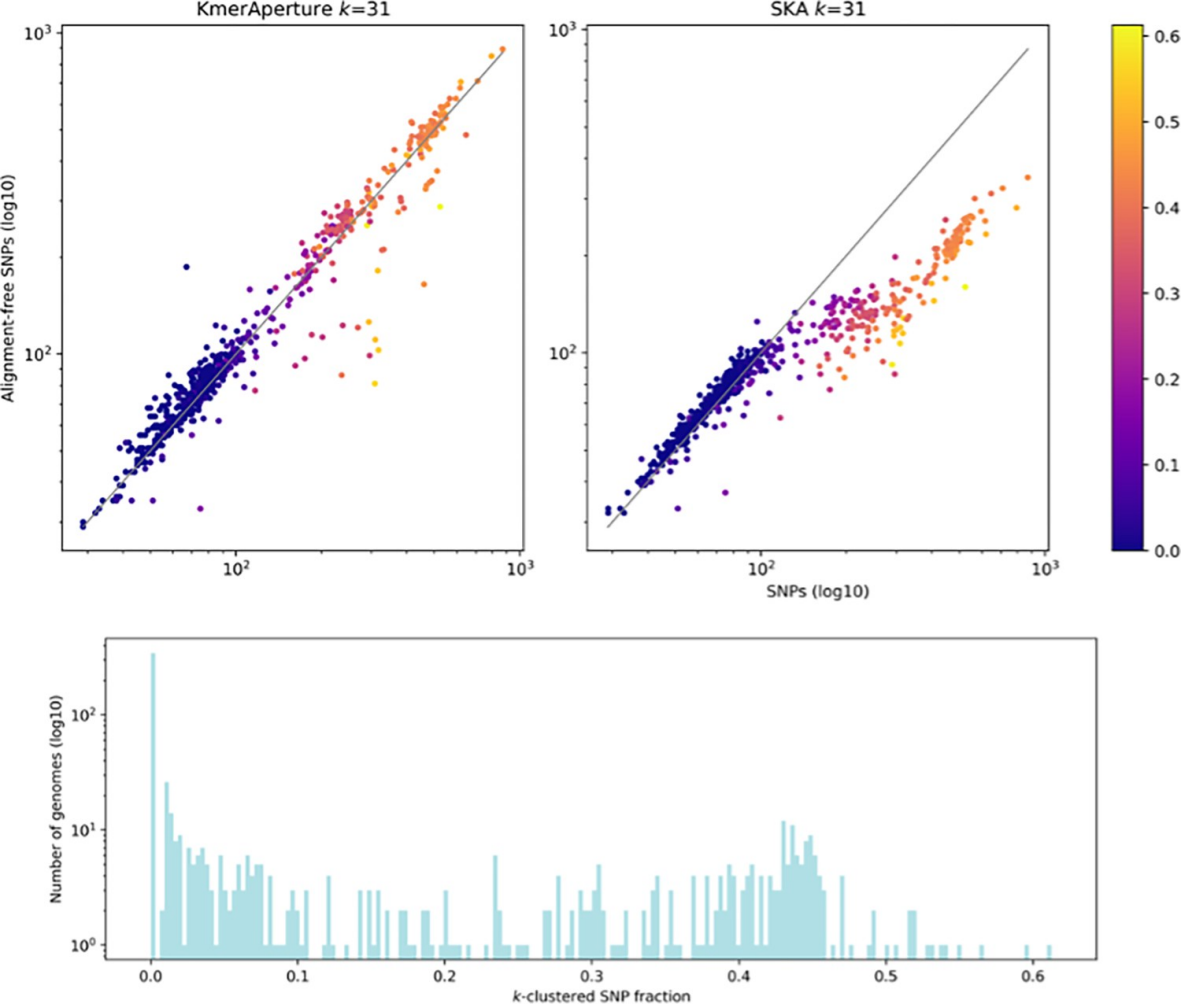
(Upper) scatterplots of *KmerAperture* (upper left) and SKA (upper right) SNPs at *k* = 31 compared with SNPs gather by snippy. Data points are coloured by the fraction of consecutive SNP pairs that are *k*-clustered for that genome. Lower, a histogram of the number of genomes with that fraction of consecutive SNPs that are *k*-clustered with *k* = 31.

The genomes in the *E*. *coli* dataset were the most diverse analysed with a median (range) of 82 (29–870) SNPs relative to the reference and large accessory differences. Of 749 *E*. *coli* genomes 476 were <100 SNPs and 53 were <50 SNPs. By comparing the *k*-mers of each *E*. *coli* genome against the reference, MCJCHV-1, *KmerAperture* predicted a median (range) of 84 (28–980), 85 (29–960) and 86 (29–892) SNPs for *k* = 19, 25 and 31 respectively. SKA produced SNP estimates with a median (range) of 117 (54–465), 93 (38–398) and 84 (32–349) for *k* = 19, 25 and 31 respectively. SKA trended towards overestimation in those with ≤150 SNPs and underestimation in those above (Fig 3). This is reflected in the SNP ranges and median estimations at *k* = 19 and 25, with an improvement on median SNPs at *k* = 31. For comparison the relationships between SNP counts and either FracMinHash, full *k*-mer sets or PopPUNK core distances are provided in S1 Fig. None of these *k*-mer set-based approaches strongly correlated with SNPs.

In this moderately diverse genome dataset, the SNPs extracted by *KmerAperture* fit the real SNP counts closely, according to the to the root-mean-squared error performance (RMSE) on log10 values. Crucially, the performance was largely independent of choice of *k*, with little difference across RMSE values. The RMSE with *KmerAperture* was 0.06 for *k* = 19 and 0.07 for *k* = 25 and 31 (S2 Fig). The level of under- and overprediction appeared to also be independent of real SNP count values. For comparison, SKA systematically overpredicted SNP counts when real values were ≤150 SNPs and underpredicted for >150 SNPs. The RMSE values were best for SKA at *k* = 25 (0.15) followed by 0.17 for *k* = 19 and 31. *KmerAperture* is designed to accurately extract SNPs within closely related genomes but the *E*. *coli* datasets shows good performance in those up to >400 SNPs (n = 95 genomes). The RMSE values for those with >400 SNPs were 0.06 for *k* = 19 and 31 and 0.08 for *k* = 25 with *KmerAperture*, whereas SKA ranged from 0.28 to 0.34. SKA map outperformed SKA align and was similar to SKA align with no *k-*mer frequency filter (S3 Fig). Additionally, SNPs were gathered with kSNP at *k* = 31 and compared to SKA map with *k* = 31. They correlated (>0.99, log10 RMSE = 0.0138) strongly with most genomes differing by 0 (n = 120), 1 (n = 217) or 2 (n = 194) SNPs (S4 Fig).

In addition to correlation to an established SNP calling method (snippy), we evaluated the level of SNP congruence with snippy SNPs treated as the ground truth. Sensitivity was high for *KmerAperture* and SKA. With *KmerAperture* sensitivity was 0.89 for *k* = 19 and 25, whilst it was 0.88 for *k* = 31. For those genomes with <100 SNPs sensitivity increased to 0.95 for *k* = 19 and 0.97 for *k* = 25 and 31. With SKA sensitivity values were 0.84, 0.79 and 0.75 for *k* = 19 to 31 respectively. These increased to 0.97 across *k* for those with <100 SNPs. The largest difference was in the number of false positives, as measured by the positive predictive value (PPV), which was 0.82, 0.83 and 0.84 for *k* = 19 to 31 respectively, whereas the PPV values for SKA were 0.35, 0.55 and 0.62 respectively. With *KmerAperture* and those <100 SNPs PPV values increased to 0.93 across *k*. SKA PPV values decreased slightly for those with <100 SNPs with 0.29, 0.52 and 0.59 for *k*19, 25 and 31 respectively.

Across the *E*. *coli* genomes, the majority had no SNPs that were *k*-clustered. At *k* = 19, 369/748 genomes had no SNPs within *k*. With *k* = 25 and 31 fewer genomes had no *k*-clustered SNPs with 356/748 and 344/748, respectively. However, significant proportions of SNPs in some genomes were *k*-clustered, i.e., within *k* of one another. For instance, at *k* = 19, 19% (147/748) of the genomes had ≥25% of consecutive SNPs being *k*-clustered. This included one genome whose SNPs were 48% 19*-*clustered (i.e., with 99/207 couples of SNPs having distance ≤19). As *k* increases in size, the number of *k*-clustered chains also increases. As such, the greatest proportion was recorded at *k* = 31, whereby 27% (201/748) of genomes had ≥25% of SNPs being *k*-clustered (Figs 3 and S5).

The minimum reference genome bases not present in the respective query genomes based on MUMmer (unaligned sequences >60bp) was 273bp and at maximum 920,525bp with a median of 47,186bp. *KmerAperture* recovered a per query genome median (range) of 46,952bp (98bp-914,782bp) with *k* = 19 increasing with *k* = 25 and 31 with a median (range) 48,746bp (210bp-921,213bp). The RMSE on log10 values reported a close fit for all values of *k* with 0.038 and 0.025 for *k* = 19 and 25 respectively and the closest fit at *k* = 31 with 0.023.

### Application to a cluster of *Salmonella* Typhimurium genomes

The *Salmonella* Typhimurium dataset (n = 1,264 genomes) represent a low diversity dataset with many highly similar genomes relative to the reference. The real SNP counts relative to the reference were median (range) 21 (1–429), with 1,176 genomes with <50 SNPs and 149 with ≤10 SNPs. *KmerAperture* was run for all genomes and extracted a median (range) of 21 (1–519), 21 (1–482) and 21 (1–462) SNPs for *k* = 19, 25 and 31 respectively. Median (range) SNP counts with SKA at *k* = 31 were 28 (3–225) SNPs with *k* = 19 and 25 with 70 (21–479) and 39 (10–300) respectively. For comparison the relationships between SNP counts and alignment-free distance estimators FracMinHash, full *k*-mer sets and PopPUNK core distances are provided in S6 Fig.

**Fig 4:**
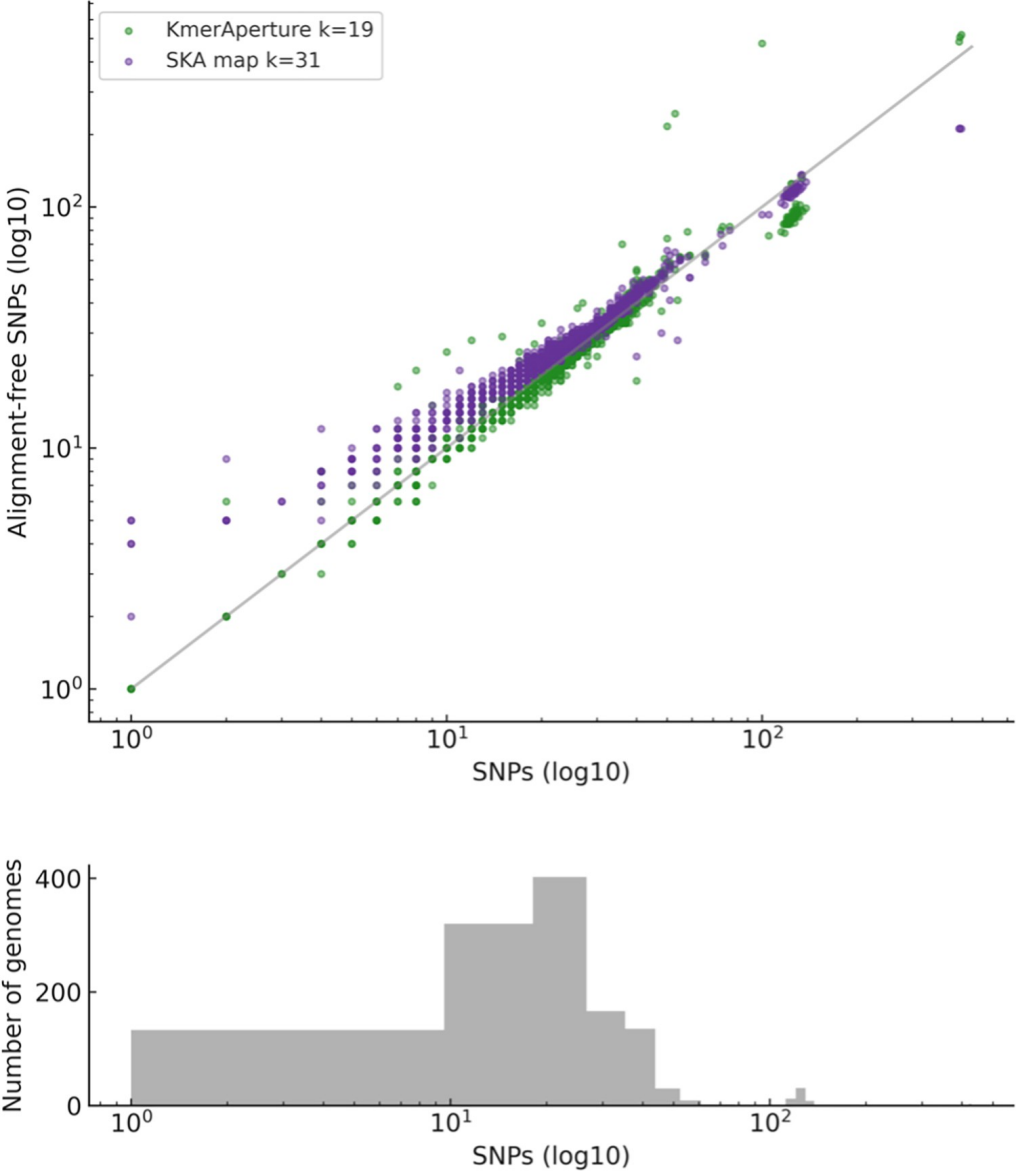
Scatterplot of *Salmonella* Typhimurium SNPs gathered by KmerAperture (green) and SKA (purple) compared with those gathered by snippy. Axes of SNP counts are log10 scaled. Line shows perfect correlation of SNP values. Values of *k* were selected based on the best correlations. Histogram shows the number of genomes for respective SNP ranges.

The SNP counts determined by *KmerAperture* fit the real SNP counts closely across choice of *k* (S7 Fig). There was similar performance with RMSE on log10 values of 0.07 for *k* = 19 and *k* = 25 and 0.08 for *k* = 31. For *k* = 31 SKA and real SNP counts RMSE was similar to *KmerAperture* with 0.09. It was sensitive of choice of *k* however, producing an RMSE of 0.27 for *k* = 25 and 0.48 for *k* = 19. SKA produced a consistent level of SNP count overprediction along the range of SNP diversity. SKA performance was best at *k* = 31, with overprediction minimised and less underprediction of the highest values relative to *KmerAperture* (Fig 4). SKA map was also compared with SKA align and kSNP. SKA map performance was highly similar to kSNP and greater than SKA align (S8 and S9 Figs).

Again, the level of SNP congruence with snippy SNPs (where they are treated as the ground truth) for SNPs gathered by *KmerAperture* or SKA. Sensitivity was high for *KmerAperture* and SKA. Sensitivity with *KmerAperture* is 0.87, 0.88 and 0.86 and PPV 0.89, 0.90 and 0.91 for *k* = 19, 25 and 31 respectively. Sensitivity values with SKA were comparatively greater with 0.95, 0.93 and 0.92 for *k* = 19, 25 and 31 respectively, though corresponding PPV values were lesser with 0.31, 0.55 and 0.73. Taking into account only those with <100 snippy SNPs (n = 1,201) sensitivity values with *KmerAperture* are 0.93, 0.95 and 0.96 for *k* = 19, 25 and 31. For SKA sensitivity is 0.97 for all *k*. PPV values for *KmerAperture* and <100 SNPs were similar with 0.92–0.93 whilst SKA PPV values reduced slightly to 0.27, 0.51 and 0.71 for *k* = 19, 25 and 31 respectively.

A flexible core genome alignment was derived from the snippy, SKA and *KmerAperture* whole genome reference-anchored alignments and the pairwise SNP counts extracted. There were 798,216 exclusive pairs with a median (range) of 34 (0–822) pairwise cgSNPs. Graphs were constructed with edges between genomes (nodes) if they met a SNP threshold of ≤2, ≤5 or ≤10 SNPs. *KmerAperture* generated graphs with 646, 941 and 1187 nodes for ≤2, <5, ≤10 respectively. These were comprised of single linkage clusters (connected components). At ≤2 SNPs, *KmerAperture* generated 145 connected components, 72 of which contained at least 3 genomes, compared with 152 real SNP clusters at ≤2 SNPs including 76 with at least 3 genomes. There was similar recovery of clusters for ≤5 and ≤10 SNPs with 82 and 34 clusters with >2 genomes with *KmerAperture* respectively compared with 80 and 37 real SNP clusters. SKA reconstructed fewer SNP clusters (>2 genomes) with 38 at ≤2SNPs, 65 at ≤5 SNPs and a greater number than with real SNPs with 65 at ≤10 SNPs.

It was determined by MUMmer that query genomes did not align to a median (range) of 45,020bp (6,917bp-216,154bp) of the reference genome. The median estimate for *KmerAperture* ranged from 43,480bp to 46,925bp for *k* = 19 and 31 respectively. The RMSE of the log10 values fit best with *k* = 25 with 0.018 and fit closely for *k* = 19 and 31 with 0.021 and 0.025 respectively (S10 Fig). This represents recovery of the majority of invariant sites between query genomes and the reference. Additionally, as with SNP counts recovered by *KmerAperture* recovery of *aligned* reference sites is independent of *k* choice with the *S*. Typhimurium dataset.

### *KmerAperture* efficiency

The runtimes for *KmerAperture* and SKA were recorded as the mean per-genome runtime by dividing the overall runtime by the number of genomes. In all cases there was a slight increase in runtime with increase in size of *k*. Mean per genome runtime with the real genome datasets was faster with *KmerAperture*. With *KmerAperture* runtime averaged between 10.41 and 13.87 seconds for the *Salmonella* Typhimurium genomes and between 13.57 and 14.82 for the *E*. *coli* genomes for *k* = 19 to *k* = 31 respectively. Whereas SKA averaged between 22.8 and 23.8 seconds for the *Salmonella* Typhimurium genomes and between 24.1 and 26.4 for the *E*. *coli* genomes for *k* = 19 to *k* = 31. Further with *KmerAperture*, query genomes may be analysed concurrently across multiple processors, making the time to extract an alignment from 1,256 *Salmonella* Typhimurium genomes (*k* = 19) 21 minutes with 10 processors, or ~1 second per genome.

Of the *E*. *coli* genomes, 10 were selected at random and line-by-line the memory used by *KmerAperture* was profiled. Peak memory usage was 1,918MiB, meaning that ~1.8Gb of RAM would be sufficient for the most memory intensive steps observed. Based on this profile concurrently running 4 genomes should be possible on a standard laptop (8Gb).

## Discussion

*KmerAperture* presents a novel alignment-free methodology which accurately determines regions of sequence similarity and SNPs within them including SNPs within *k* of one another, a constant problem for *k*-mer based solutions. Though we intend to compare closely related genomes, unevenly spatially distributed SNPs are not unusual. In the *E*. *coli* genome sample at *k* = 31 27% (201/748) of genomes had > 25% of their SNPs ≤31bp apart. Another important development is the insensitivity to choice of *k*. When these methods are applied, the ground-truth SNPs are not known and as such lack a meaningful approach to selecting *k*.

In the examples tested *KmerAperture* works as well, in situations where genomes differ by <50 SNPs, as those with >400. At present, methods such as MinHash may cluster genomes into MLST-level groups. Natural populations of bacteria, separated by deep branches in the species are well reflected by MLST. Within MLST-defined clusters may involve differences in the thousands of SNPs. SKA, the best alignment-free methodology for determining SNPs in closely related bacteria, is intended for application to much less diversity such as transmission chains. This means however, that we also need a method that can first determine groups of this level of diversity from within a sequence type. *KmerAperture* is also able to properly determine similarity at this level.

We implemented *KmerAperture* as reference-based and as is comparable to SKA map. This decision was based on the benefits of a linearly increasing number of runs with additional genomes. The underlying algorithm, however, is not limited to being reference-based. We hope that this new approach to alignment-free comparisons will be useful but that also the new framework of discerning diversity containing *k*-mers may provide the basis for further development. A current limitation is that assembled genomes are required as input. SKA, for example, can also take unassembled reads as input.

The *KmerAperture* algorithm is based on the few axioms we have of how relative sequence differences are represented by comparing *k*-mers. In sequences of the same length, with >*k* spaced SNP positions and no other variation, the number of unique *k*-mers per genome / *k* will represent the number of variant positions. In reality, bacterial genomes have large unaligned regions, *k*-clustered SNPs, indels, structural rearrangements, repetitions and other complexities. We demonstrated with simulated and real datasets that by retaining *k*-mer synteny we can accurately recover SNPs, including many of those that are *k*-clustered. Initially the algorithm filters mismatched *k*-mers by their original order, nominating contiguous series of length *k* as being generated by a single SNP difference between the genomes. This is followed, by matching the middle *k*-mers without the middle base. When several SNPs’ positions occur ≤*k* bp from each other, series of length >*k* are generated. By pattern matching between same length series, we explicitly consider this possibility. The persistent problem in *k*-mer based alignment-free analyses is here resolved. Essentially, *KmerAperture* foregoes alignment as matching flanking regions to *k*-mer series are implicit in their presence in the *k*-mer set intersection, which can be rapidly determined. With *KmerAperture*, the entire relative diversity of genomes is encoded in the syntenic *k*-mer series. Methods may be improved further to types of diversity such as indels and sequence rearrangements.

Since *KmerAperture* determines the position of each SNP, it can be used to generate a reference-anchored alignment containing all the SNPs found in a set of genomes of interest. Unlike a simple SNP matrix with no notion of SNP locations, this *KmerAperture* output can be to used to perform a recombination-aware phylogenetic inference, for example using ClonalFrameML [21] or Gubbins [22]. In this context, it is especially important that *KmerAperture* attempts to reconstruct SNPs within *k* bp of each other, since bacterial homologous recombination, especially when the donor and recipient are distantly related, has a high potential to generate such clusters of SNPs [23].

Unlike other tools that are focused on SNP detection, *KmerAperture* is also able to identify accessory regions, and may therefore be developed for pan-genome analyses [24]. It would also be possible to extract accessory sequence positions, prior to alignment. The computational and temporal challenge of alignment is related both to the number of sequence comparisons but also the diversity of the sequences under study. Reducing this diversity, especially in genome samples of unknown diversity, would reduce the cost of alignment. *KmerAperture* could also be adapted to progressively remove accessory regions as genomes are added, compare the genomes to a core genome alignment, or to a reference.

It could next be examined whether clustering on these SNPs, or construction of a phylogenetic tree from the alignment may be used as a scalable alternative to sub-MLST lineage discrimination. For instance, clustering the *S*. Typhimurium genomes by SNP thresholds suggest that this may be a viable tool to test on numerous species, to provide results comparable to alignment. This is crucial because whilst existing tools may be accurate once very similar genomes have been identified, this is the first alignment-free methodology able to discern clusters of very similar genomes, from those that are moderately similar (~<1,000 SNPs).

The datasets were chosen to reflect real-life scenarios of the type of diversity according to which we might cluster genomes, either by genomic typing or by methods, such as MinHash, not involving alignment. Divergent genomes can be identified and removed, retaining only the low-diversity genomes to be analysed further, possibly using alignment-based comparison. In a scenario where there is a survey of bacterial genomes, for instance in a clinical setting, it is important to rapidly differentiate species, lineage, sub-lineage and possible outbreak clusters based on genetic distance. The size and diversity of these datasets can render alignment-based methods difficult and possibly intractable unless an excess amount of computational resources is used. *KmerAperture* runs in a few seconds on a laptop, and genomes may be compared concurrently, making it a scalable approach for genomic surveillance.

## Methods

### Using synteny to distinguish core and accessory variation

The data for analysis consists of a single reference genome and several query genomes, all of which have been assembled *de novo* and may therefore be made of multiple contigs [25]. Both reference and query genomes are decomposed into *k*-mers and canonicalised, which involves reverse complementing each *k*-mer, comparing to the original and selecting the lesser *k*-mer. The *k*-mer window size (*k*) is defaulted to 19, whilst various other *k* sizes were also explored.

For each query genome the full set of *k*-mers is constructed for the query and reference (sets *R* and *Q* respectively). Next the relative complement of each set (*R*^*c*^ and *Q*^*c*^) are determined by subtracting the sets from one another. The original lists of *k*-mers (which unlike the sets are not deduplicated) were enumerated. The *k*-mers of the relative complements were then matched back to their respective *k*-mer lists to derive their synteny. Each matched *k*-mer position is then stored and sorted as ranges or *k*-mer ‘series’. The length of these series is then used to evaluate the underlying comparative diversity that has generated these mismatching *k*-mers.

Series of *k*-mers that equal *k* in length (*L*) are provisionally taken to be the result of a SNP, whilst those greater than *k* in length are stored as accessory/other. It was enforced that *k* must be an odd number so that the middle *k*-mer may be extracted from all series of length *k*, the middle base removed and then the number of *k*-mers that would match without the middle base counted.

Whether there are SNPs within *k* of one another is evaluated next, including accounting for the possibility of chaining of multiple SNPs within *k*. Series of length *L* ranging from *k*+2 to *S*(*k*-1)+1 have their original sequences reconstructed, where *S* is the maximum number of ‘chained’ SNPs (defaulted to 10). Unlike with single SNPs there is not an explicit relationship expected between *L* and number of polymorphisms. Instead, all possible pairs of the same *L* are compared including the respective reverse complement pairings, mismatching bases counted and a filter of ≤25% divergence applied. All mismatches must not be contiguous in the sequence, with spacing of at least 1bp required.

### Implementation and benchmarking of *KmerAperture*

*KmerAperture* is written in python 3 and depends on the python packages numpy [26], biopython [27] and the screed module of the khmer package [28]. An OCaml, compiled, parser processes input genomes in a computationally efficient manner, decomposing them into *k*-mers and canonicalising them. Visualisations were generated in python with the use of seaborn [29] and matplotlib [30] in the Jupyter Notebook environment [31]. Conda was used for package management [32]. *KmerAperture* was compared with SKA *map* SNP counts. SKA *align* was also run for comparison, the pairwise option of SKA. SKA *map* was chosen as the primary comparator as it’s analogous to our reference-based implementation of *KmerAperture*. We ran *KmerAperture* on assembled genomes across a range of values of *k* (*k* = 19, 25 and 31), where *k* must be an odd number. SKA requires that the *k* value be generated by an integer divisible by 3, that represents the length of each sequence flanking a flexible base. As such, a range of flanking lengths was selected of 9, 12, and 15 to generate the same *k* = 19, 25, and 31 as in *KmerAperture*. Real genome SNP counts were derived from the tool snippy [33], whilst unshared sequences were determined by MUMmer dnadiff [34]. Additionally, for comparison, it was assessed to what extent full *k*-mer sets Jaccard distances correlate with SNPs. We further compared SNPs to PopPUNK, a MinHash sketch-based approach for discerning within-lineage distances. SKA was also compared to kSNP4 [16]

### Data acquisition and processing

The reference genome *Klebsiella pneumoniae* HS11286 [NC_016845, 35] was downloaded and used as the template for each simulated genome. Two sets of simulated genomes were constructed: in the first set genomes relative to the reference have additional sequence and SNPs and in the second set all genomes are the same size as the reference with SNPs only.

For the first set, 500 genomes were simulated with between 1–100 SNPs and 1–30 accessory sequences of 1,000–10,000bp. The number of accessory sequences, the location where they were inserted and the genome positions to mutate, were all uniformly sampled at random whilst the number of SNPs were sampled from a log10 distribution. There was no relationship simulated between amount of accessory sequence and number of SNPs. All genomes were randomly assigned between 1,000 and 300,000bp of accessory sequence.

For the second set, genomes were simulated with SNPs relative to the reference only. Five subsets, each with 100 genomes were constructed. All genomes had 50 SNPs relative to the reference. The five subsets were constructed to contain 20%, 40%, 60%, 80% and 100% of the SNPs (n = 10,20,30,40,50 SNPs respectively) to be within *k* of at least one other SNP. For each genome it was randomly determined how many SNPs, up to 10, would be within *k* and how large the spacing between each SNP was, up to *k*. The size of *k* was fixed to 25.

Real genome datasets were also used to assess *KmerAperture* performance, a low diversity and medium diversity within-lineage population. The first was a medium diversity cluster of genomes, subset of the pandemic *Escherichia coli*, MLST-defined cluster ST1193. The ST1193 population records were further subset to the HierCC [36] HC20-level cluster 571 (n = 1,331), where they were filtered for those with NCBI ‘biosample’ identifiers (n = 1,233), whereby 757 assemblies were successfully downloaded. Finally, 8 genomes were removed for being too diverse (>1,000 SNPs including 6 at >30,000) resulting in 749 for analysis. Within cluster HC20 level cluster 571, reference genome *E*. *coli* MCJCHV-1 sequence (chromosomal: CP030111.1), was selected for mapping and variant calling. EnteroBase [5] was accessed and searched for these criteria on 22/02/2023.

The second cluster of real genomes was a subset of the *Salmonella enterica* subsp. *enterica* Serovar Typhimurium (*Salmonella* Typhimurium) MLST defined cluster 34 (ST34). The ST34 population records were further subset to the HierCC [36] HC5-level cluster 302 (HC302, n = 2,399). Within cluster HC302 cluster, the completely assembled genome *S*. Typhimurium S04698-09, was selected as the reference. EnteroBase [5] was accessed and searched for these criteria on 22/02/2023. All genomes with NCBI biosamples were selected for download (n = 1,768), of which 1,283 successfully downloaded. Further, 3 genomes were excluded due to being too diverse (>300 SNPs) whilst 16 were removed for containing non-{ATCGN} bases. Finally, 8 genomes were removed for reporting an MLST other than ST34, resulting in the final set of 1,256 genomes.

All genomes were downloaded from the NCBI genomes database (S1 Table).

## Supporting information

S1 Table Genome assemblies downloaded from Enterobase and their corresponding NCBI accessions. (CSV)

S2 Table Performance by SNP retrieval for *KmerAperture* and SKA. There were three SNP amounts (50, 100, 150) and five fractions of *k*-clustering for each (20, 40, 60, 80 and 100% density). Within each category 50 genomes were simulated. The median retrieval is reported for each category with individual genome minimum and maximum retrieval.
(CSV)

S1 Fig Scatterplots of SNPs vs alignment free distance estimations for 749 *E*. *coli* genomes against the reference (MCJCHV-1 chromosome). Of these 476 were <100 SNPs and 53 were <50 SNPs. Plots to the right are log10 scaled. In blue (first row) are the distances from PopPUNK *core* with a sketch size of 10e6 and in teal (second row), the PopPUNK accessory distances. Unadjusted *k*-mer Jaccard distances vs SNPs are magenta (third row). The unadjusted *k*-mers are canonical *k*-mer set 1-Jaccard values.
(TIF)

S2 Fig Scatterplots of SNPs gathered by snippy and *KmerAperture* for 749 *E*. *coli* genomes against the reference (MCJCHV-1 chromosome). Each plot is presented on the left without scaling and on the right with log10 scaling. The plots (upper to lower) are of *KmerAperture* with *k* = 19, 25 and 31 respectively.
(TIF)

S3 Fig Scatterplots of SNPs gathered by snippy and SKA for 749 *E*. *coli* genomes against the reference (MCJCHV-1 chromosome). Each plot is presented on the left without scaling and on the right with log10 scaling. All plots are for *k* = 31. The plots (upper to lower) are of SKA align with no split *k*-mer frequency filtering, SKA align default, SKA map default and SKA map with repeat split *k*-mers also mapped.
(TIFF)

S4 Fig The number of SNPs in the referenced anchored *Escherichia coli* alignments of SKA map against kSNP. (TIF)

S5 Fig For 749 *E*. *coli* genomes compared with the reference (MCJCHV-1 chromosome), a scatterplot of the number of consecutive SNP pairs against the number which are *k*-clustered. This was performed for *k* = 19 (blue), 25 (red) and 31 (green).
(TIF)

S6 Fig Scatterplots of SNPs vs alignment free distance estimations for 1,264 *S*. Typhimurium genomes against the reference. Of these 1,176 were <50 SNPs and 149 were <10 SNPs. Plots to the right are log10 scaled. In blue (first row) are the distances from PopPUNK *core* with a sketch size of 10e6 and in teal (second row), the PopPUNK accessory distances. Unadjusted *k*-mer Jaccard distances vs SNPs are magenta (third row). The unadjusted *k*-mers are canonical *k*-mer set 1-Jaccard values.
(TIF)

S7 Fig Scatterplots of SNPs gathered by snippy and *KmerAperture* for 1,264 *S*. Typhimurium genomes against the reference. Each plot is presented on the left without scaling and on the right with log10 scaling. The plots (upper to lower) are of *KmerAperture* with *k* = 19, 25 and 31 respectively.
(TIF)

S8 Fig Scatterplots of SNPs gathered by snippy and SKA for 1, 264 *S*. Typhimurium genomes against the reference (chromosome only). Each plot is presented on the left without scaling and on the right with log10 scaling. All plots are for *k* = 31. The plots (upper to lower) are of SKA align with no split *k*-mer frequency filtering, SKA align default, SKA map default and SKA map with repeat split *k*-mers also mapped.
(TIF)

S9 Fig kSNP vs SKA. The number of SNPs in the referenced anchored *Salmonella* Typhimurium alignments of SKA map against kSNP. Genomes were randomly selected for analysis (n = 500) due to the full set taking over 48 hours (server limit).
(TIF)

S10 Fig *Salmonella* Typhimurium number of reference sites not present in query genomes determined by *KmerAperture* compared with unaligned reference bases with MUMmer, at *k* = 19, 25 and 31. (TIF)
