# Supplementary figures and images for: KmerAperture: Retaining k-mer synteny for alignment-free extraction of core and accessory differences between bacterial genomes
Source: PLoS Genet. 2024 Apr 29;20(4):e1011184. doi: 10.1371/journal.pgen.1011184 (PMC11101094; doi:10.1371/journal.pgen.1011184)

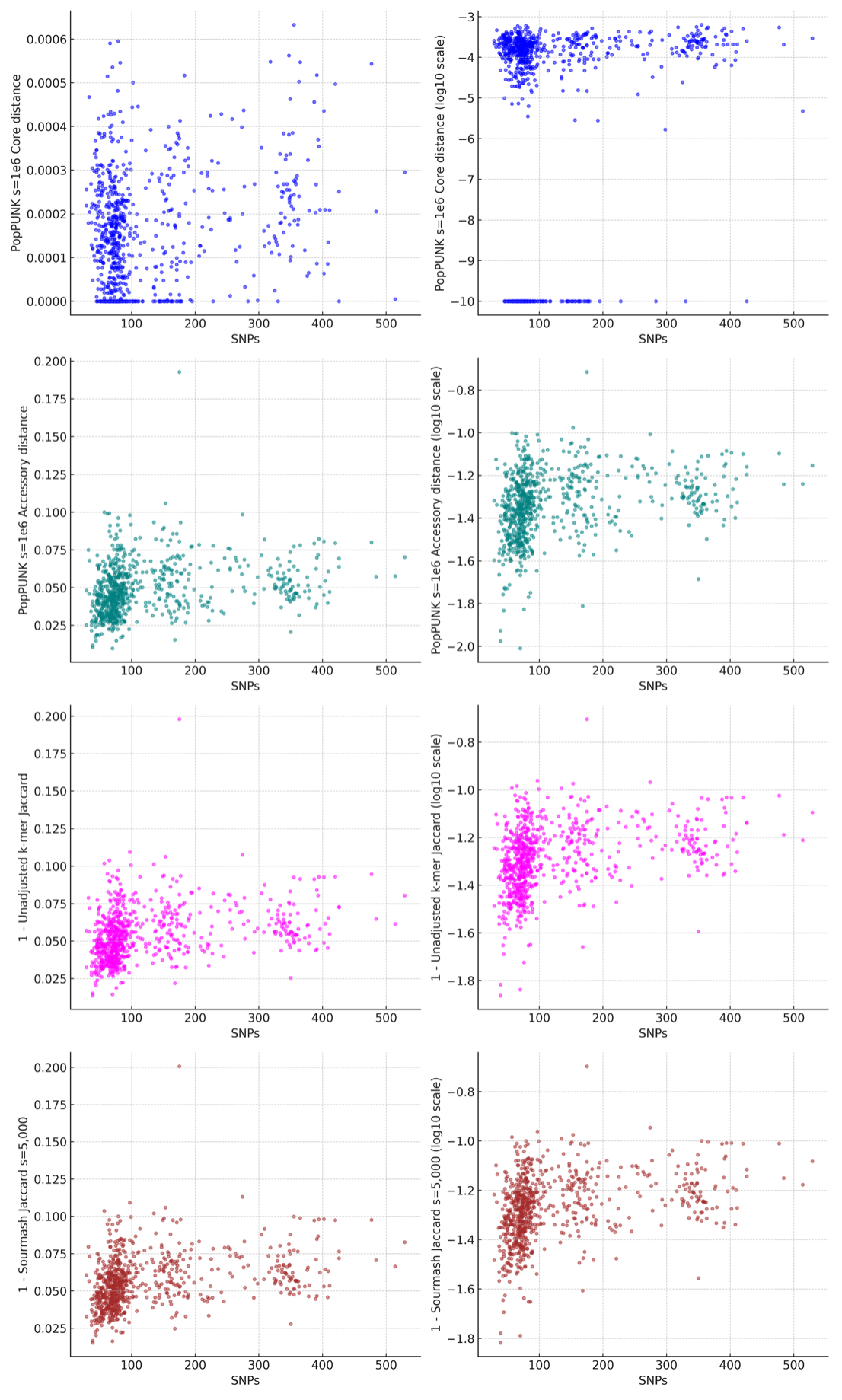

Supplement: S1 Fig — Of these 476 were <100 SNPs and 53 were <50 SNPs. Plots to the right are log10 scaled. In blue (first row) are the distances from PopPUNK core with a sketch size of 10e6 and in teal (second row), the PopPUNK accessory distances. Unadjusted k-mer Jaccard distances vs SNPs are magenta (third row). The unadjusted k-mers are canonical k-mer set 1-Jaccard values. (TIF) [file pgen.1011184.s003.tif]

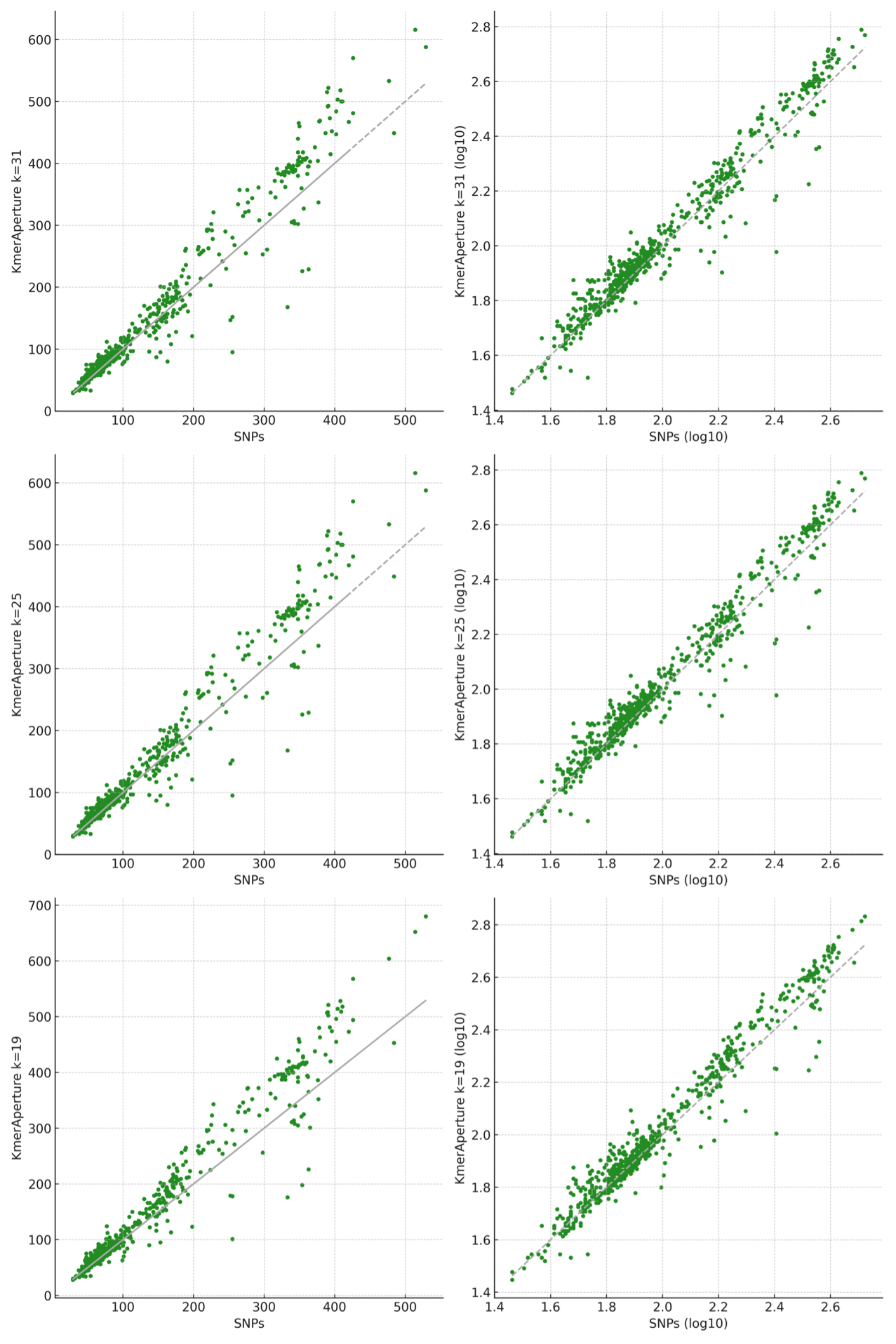

Supplement: S2 Fig — Each plot is presented on the left without scaling and on the right with log10 scaling. The plots (upper to lower) are of KmerAperture with k = 19, 25 and 31 respectively. (TIF) [file pgen.1011184.s004.tif]

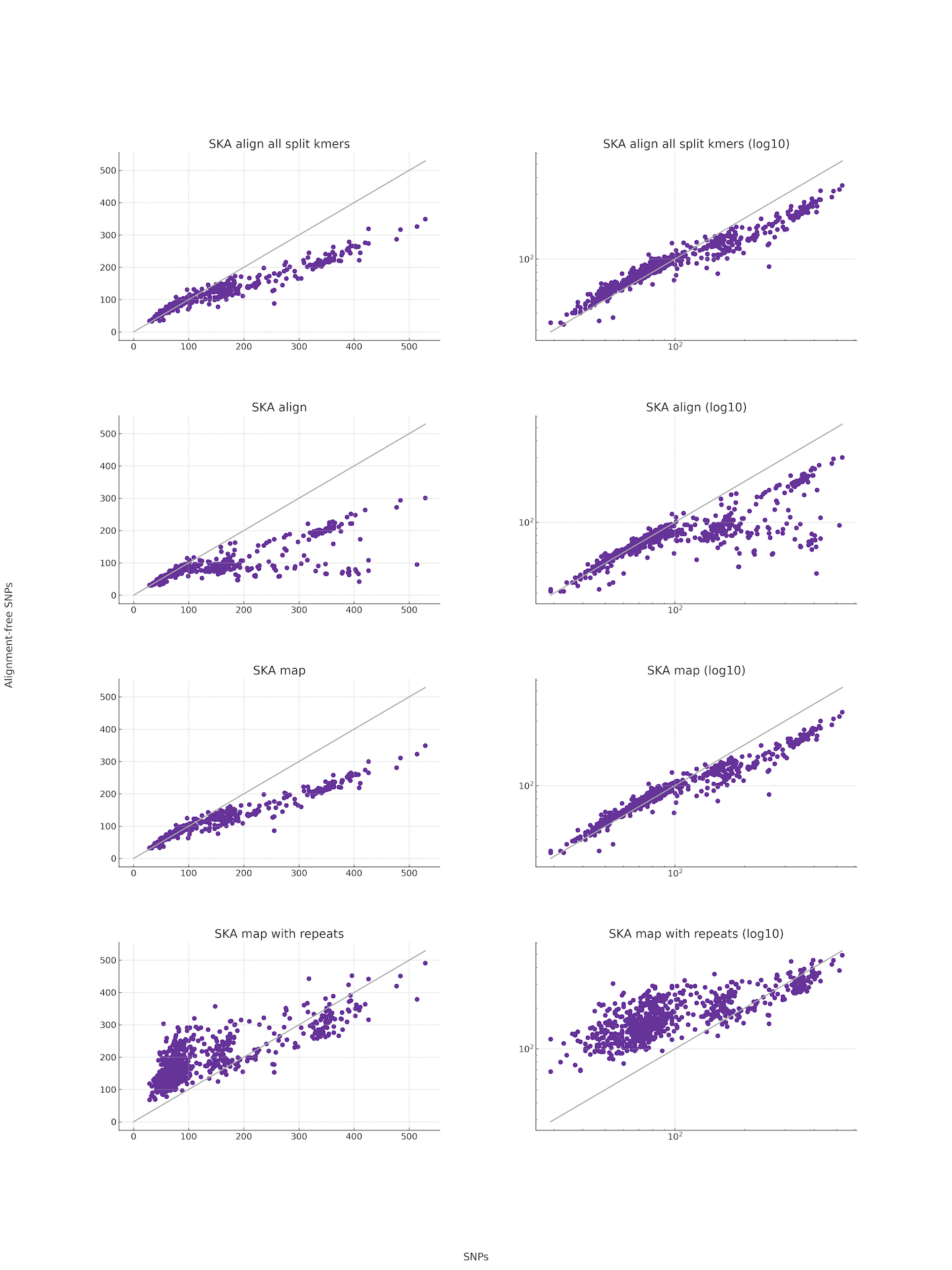

Supplement: S3 Fig — Each plot is presented on the left without scaling and on the right with log10 scaling. All plots are for k = 31. The plots (upper to lower) are of SKA align with no split k-mer frequency filtering, SKA align default, SKA map default and SKA map with repeat split k-mers also mapped. (TIFF) [file pgen.1011184.s005.tiff]

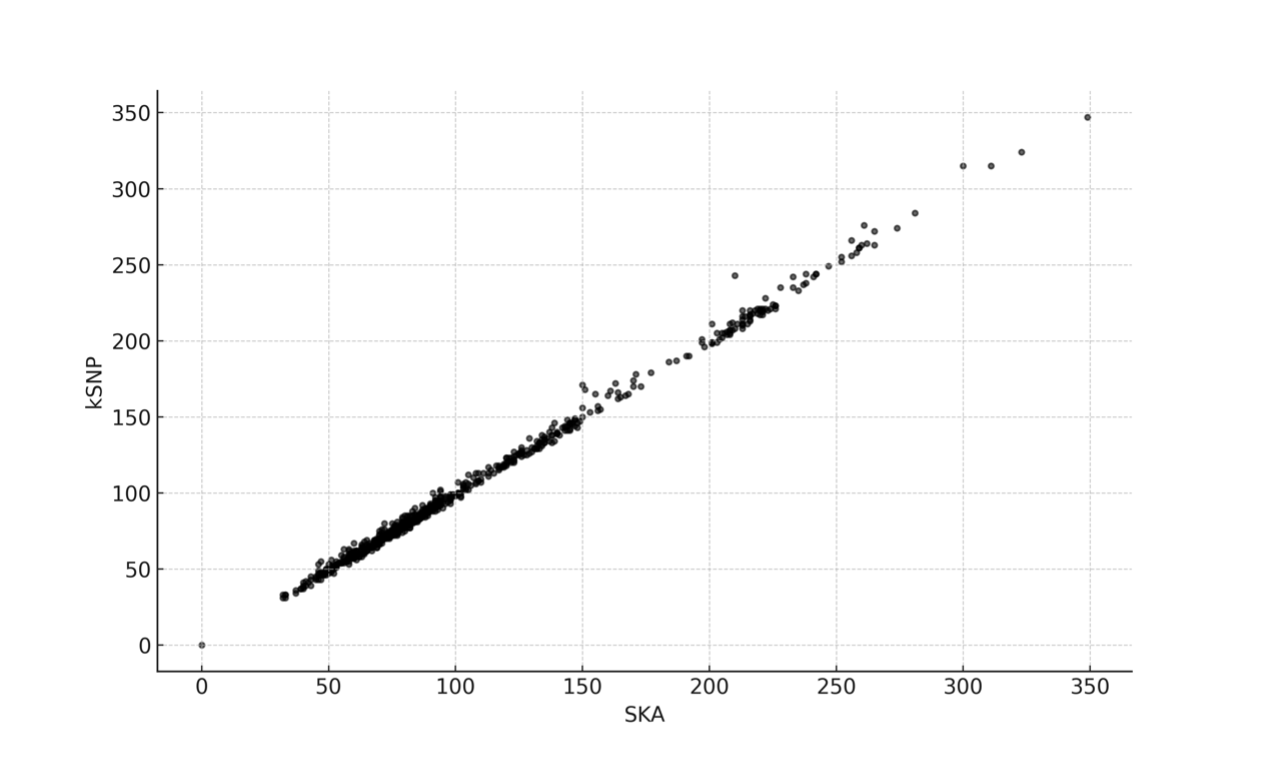

Supplement: S4 Fig — (TIF) [file pgen.1011184.s006.tif]

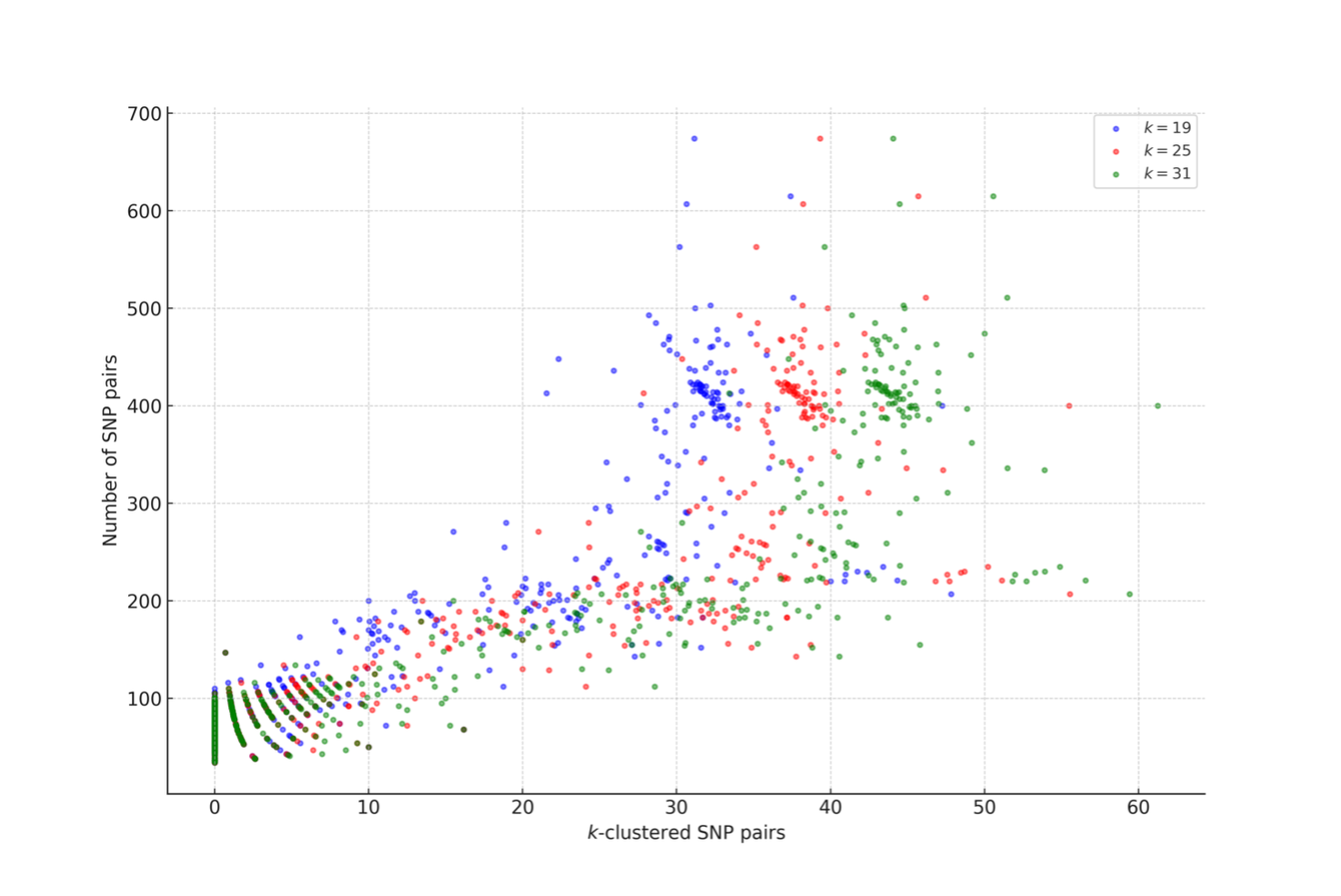

Supplement: S5 Fig — This was performed for k = 19 (blue), 25 (red) and 31 (green). (TIF) [file pgen.1011184.s007.tif]

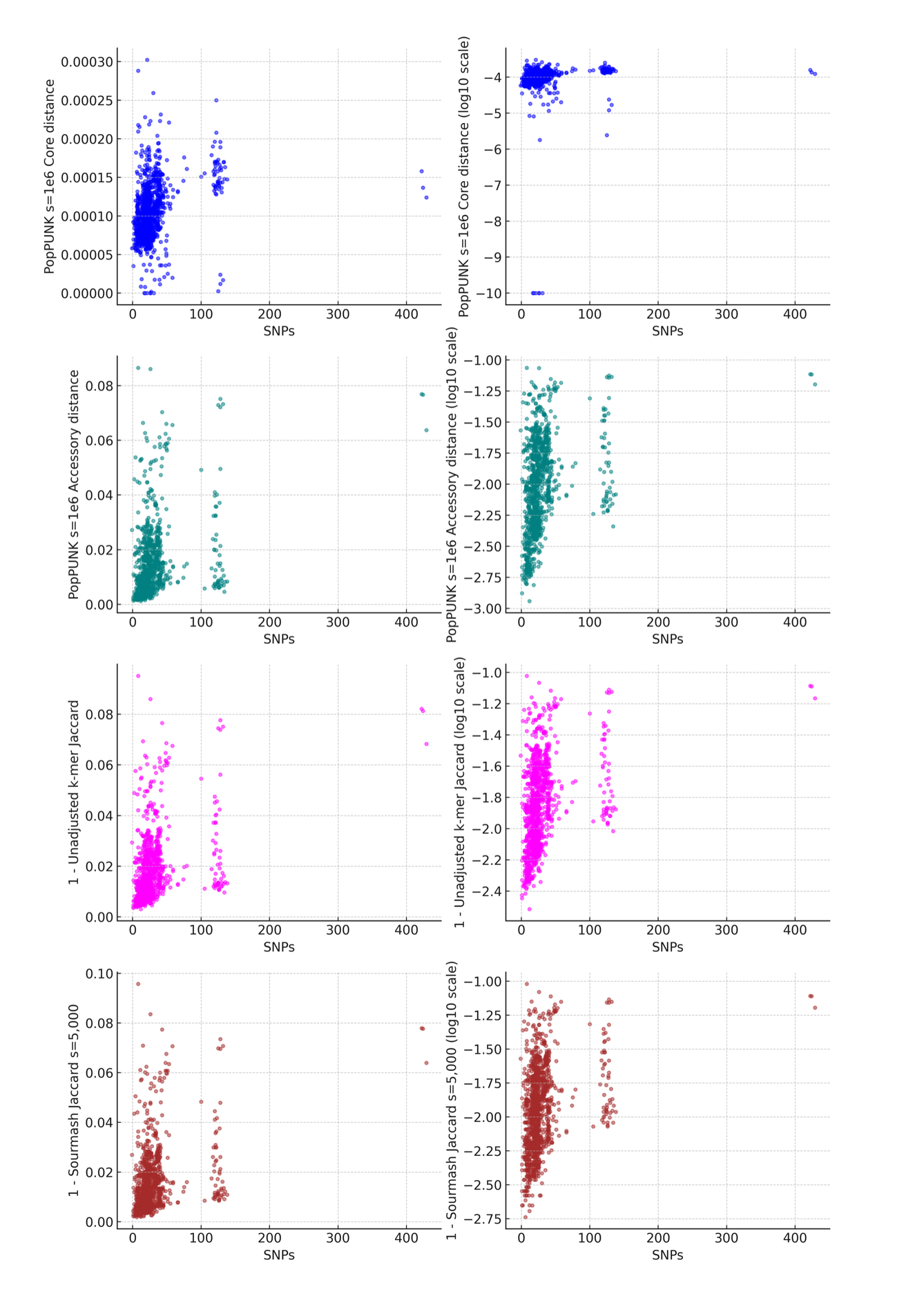

Supplement: S6 Fig — Of these 1,176 were <50 SNPs and 149 were <10 SNPs. Plots to the right are log10 scaled. In blue (first row) are the distances from PopPUNK core with a sketch size of 10e6 and in teal (second row), the PopPUNK accessory distances. Unadjusted k-mer Jaccard distances vs SNPs are magenta (third row). The unadjusted k-mers are canonical k-mer set 1-Jaccard values. (TIF) [file pgen.1011184.s008.tif]

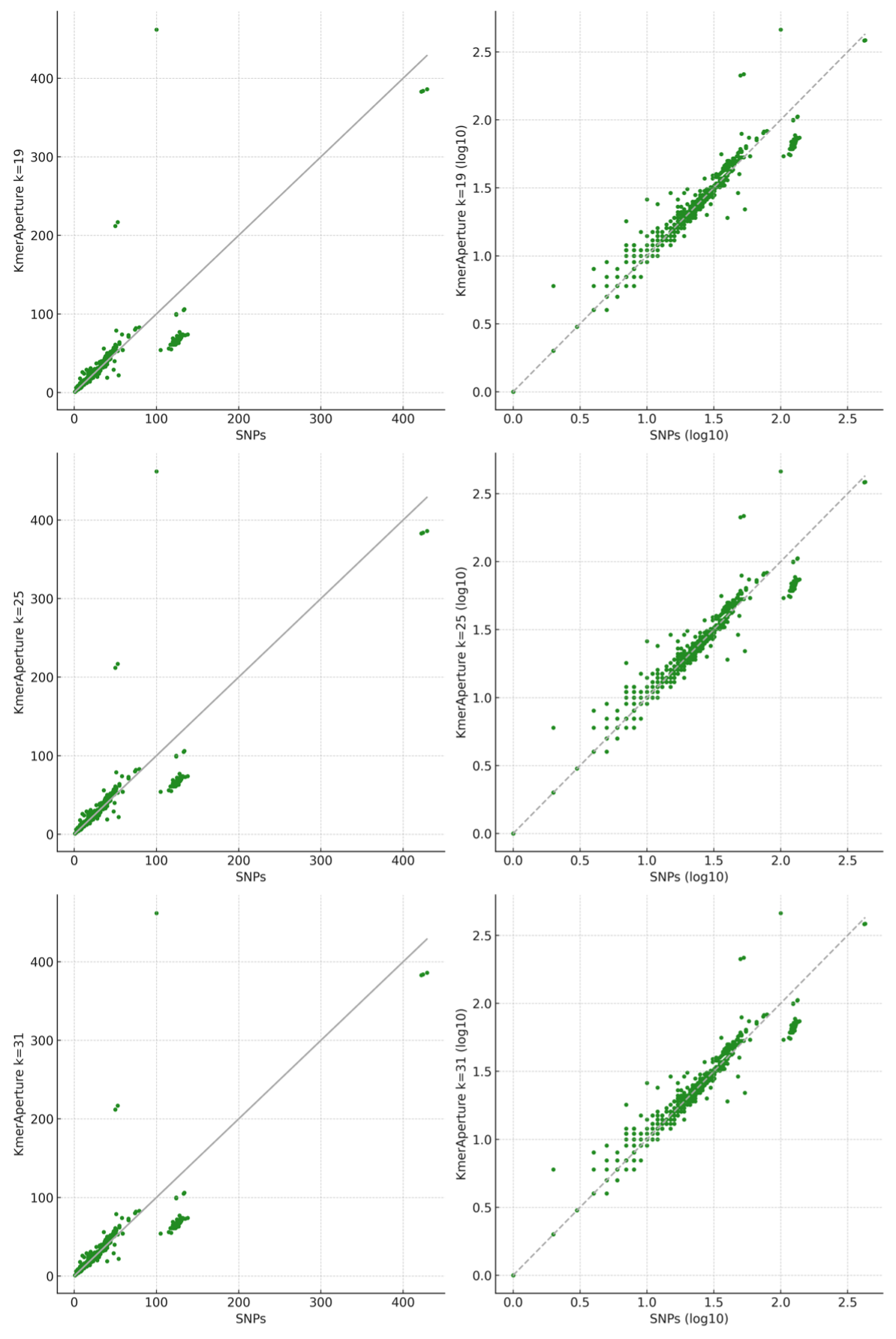

Supplement: S7 Fig — Each plot is presented on the left without scaling and on the right with log10 scaling. The plots (upper to lower) are of KmerAperture with k = 19, 25 and 31 respectively. (TIF) [file pgen.1011184.s009.tif]

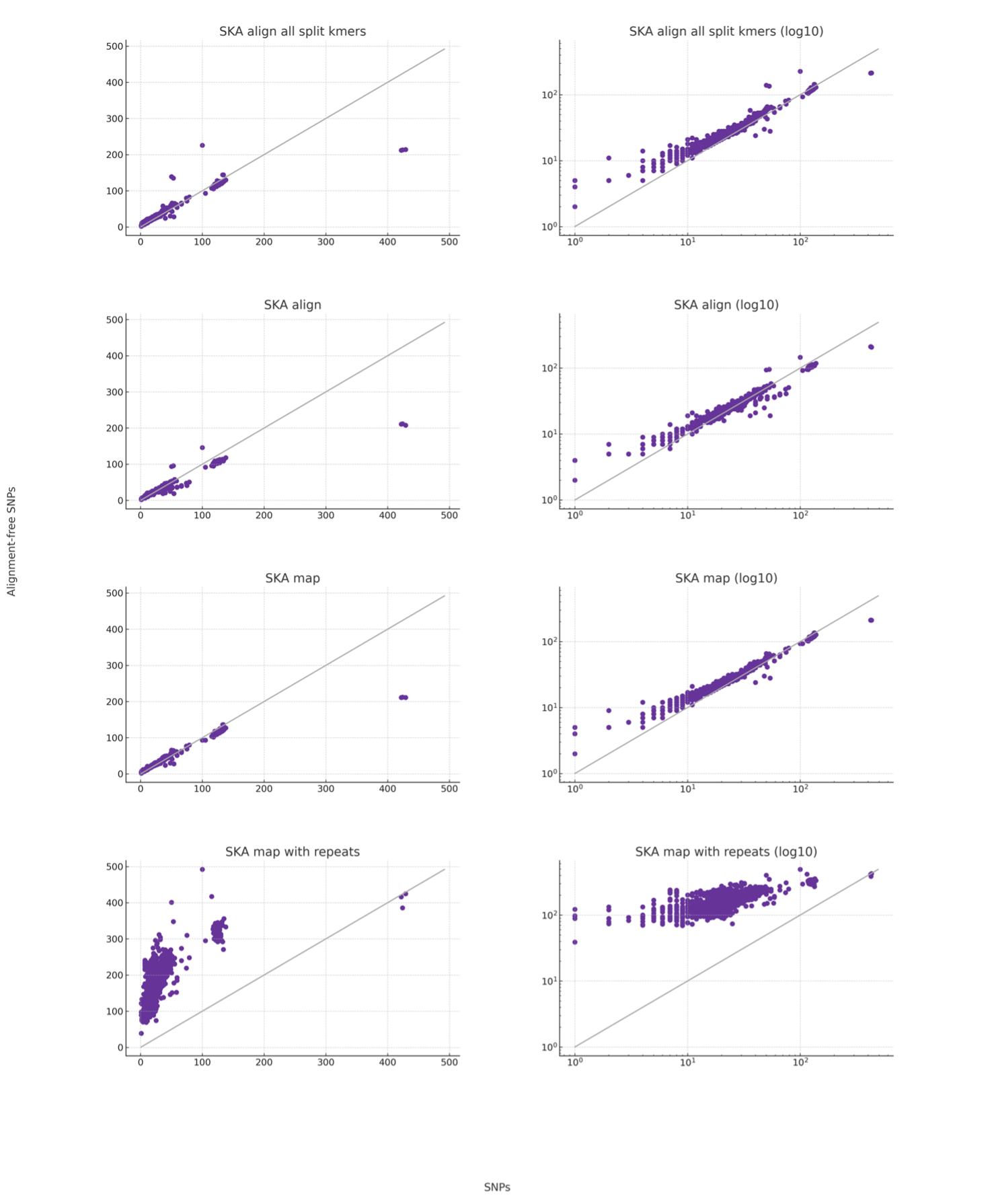

Supplement: S8 Fig — Each plot is presented on the left without scaling and on the right with log10 scaling. All plots are for k = 31. The plots (upper to lower) are of SKA align with no split k-mer frequency filtering, SKA align default, SKA map default and SKA map with repeat split k-mers also mapped. (TIF) [file pgen.1011184.s010.tif]

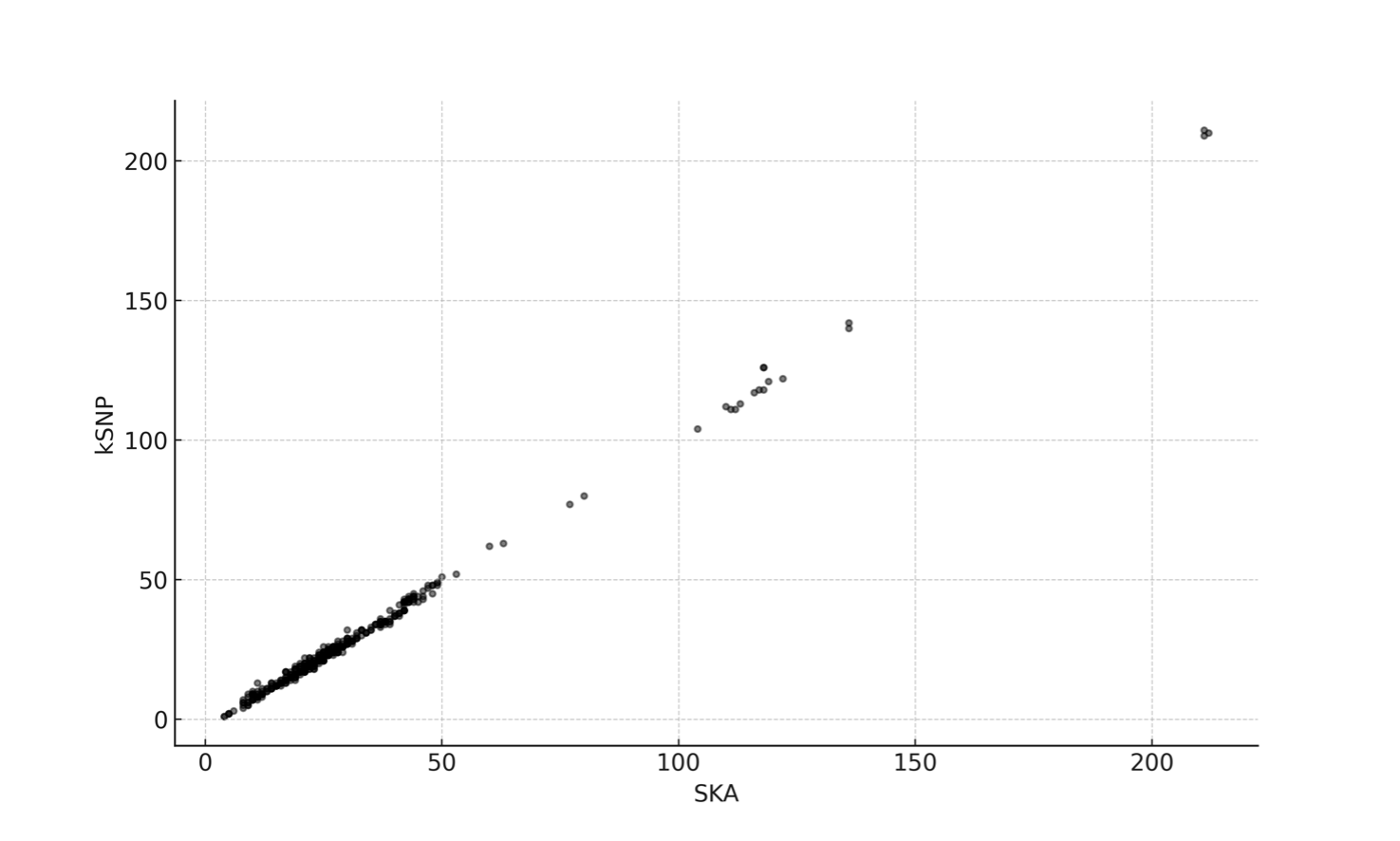

Supplement: S9 Fig — The number of SNPs in the referenced anchored Salmonella Typhimurium alignments of SKA map against kSNP. Genomes were randomly selected for analysis (n = 500) due to the full set taking over 48 hours (server limit). (TIF) [file pgen.1011184.s011.tif]

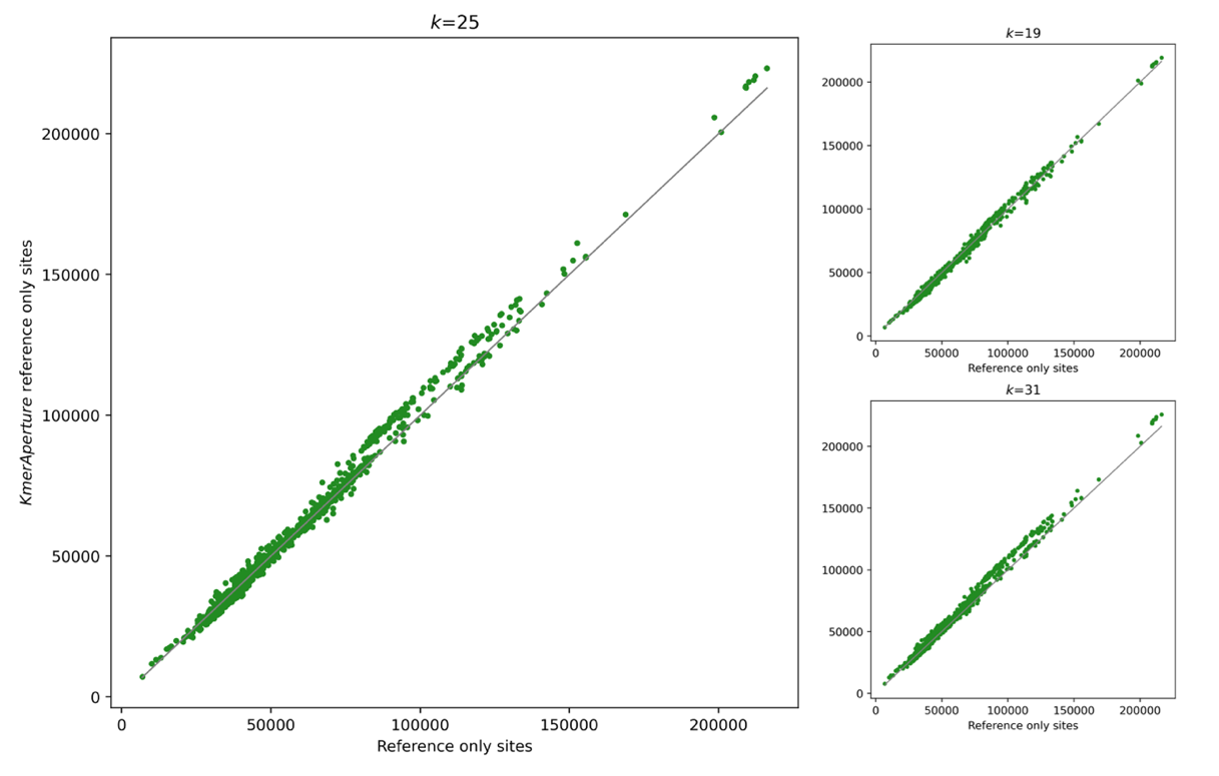

Supplement: S10 Fig — (TIF) [file pgen.1011184.s012.tif]
